# Treatment patterns and sequences of pharmacotherapy for patients diagnosed with depression in the United States: 2014 through 2019

**DOI:** 10.1186/s12888-019-2418-7

**Published:** 2020-01-03

**Authors:** David M. Kern, M. Soledad Cepeda, Frank Defalco, Mila Etropolski

**Affiliations:** 1 Janssen Research & Development, Epidemiology, Titusville, NJ 08560 USA; 2 Janssen Research & Development, Neuroscience TA, Titusville, NJ 08560 USA

**Keywords:** Depression, Treatment patterns, Antidepressants, Real-world evidence

## Abstract

**Background:**

Understanding how patients are treated in the real-world is vital to identifying potential gaps in care. We describe the current pharmacologic treatment patterns for the treatment of depression.

**Methods:**

Patients with depression were identified from four large national claims databases during 1/1/2014–1/31/2019. Patients had ≥2 diagnoses for depression or an inpatient hospitalization with a diagnosis of depression. Patients were required to have enrollment in the database ≥1 year prior to and 3 years following their first depression diagnosis. Treatment patterns were captured at the class level and included selective serotonin reuptake inhibitors (SSRIs), serotonin and norepinephrine reuptake inhibitors, tricyclic antidepressants, other antidepressants, anxiolytics, hypnotics/sedatives, and antipsychotics. Treatment patterns were captured during all available follow-up.

**Results:**

We identified 269,668 patients diagnosed with depression. The proportion not receiving any pharmacological treatment during follow-up ranged from 29 to 52%. Of the treated, approximately half received ≥2 different classes of therapy, a quarter received ≥3 classes and more than 10% received 4 or more. SSRIs were the most common first-line treatment; however, many patients received an anxiolytic, hypnotic/sedative, or antipsychotic prior to any antidepressive treatment. Treatment with a combination of classes ranged from approximately 20% of first-line therapies to 40% of fourth-line.

**Conclusions:**

Many patients diagnosed with depression go untreated and many others receive a non-antidepressant medication class as their first treatment. More than half of patients received more than one type of treatment class during the study follow up, suggesting that the first treatment received may not be optimal for most patients.

## Background

Major depressive disorder is highly prevalent in the United States, affecting more than 7% of adults and 13% of adolescents [1]. Treatment options for depression include psychotherapy, pharmacotherapy, transcranial magnetic stimulation (TMS) and invasive treatments such as electroconvulsive therapy (ECT) [2]. Treatment algorithms exist which aim to provide guidance to physicians in making effective treatment decisions to improve the chances of achieving a response [3, 4]; however, these algorithms do not provide granularity about specific medications to prescribe as there are many different treatment choices that fit within the algorithm structure, though starting with a selective serotonin reuptake inhibitor (SSRI) is the most commonly recommended approach. For patients treated with pharmacotherapy, only one quarter achieve full response and remission, while a similar proportion achieve no response, and the remainder achieve partial response or response without remission [2].

Currently, there is a lack of detail about how patients diagnosed with depression are treated in the real-world. Understanding treatment patterns in a real-world setting, as opposed to trials in which the patterns are predetermined [5], is an important step to improving and understanding gaps in care. For example, atypical antipsychotics are the only approved adjunctive therapy for the treatment of depression [6, 7], but it is unknown how often these are used or how often other off-label adjunctive treatments are used.

This paper aims to fill the gaps in knowledge about real-world pharmacologic treatment patterns of patients diagnosed with depression and the role adjunctive therapy plays.

## Methods

### Depression cohort

We identified patients diagnosed with depression from large national insurance claims databases during 1/1/2014 through 1/31/2019. We chose the most recent 5 years of data, rather than a larger time period using all available data, in order to capture a snapshot of current treatment practices rather than historical treatment practices which may no longer be prevalent. More detail about the databases is found in the ‘Data Source’ section below. Depression was defined according to the algorithm validated by Solberg et al. [8] which required the presence of two outpatient diagnoses of depression within 365 days of each other or one inpatient depression diagnosis according to the ICD-9-CM codes (269.2*, 269.3*, 300.4, 311) or the corresponding ICD-10-CM codes (F32.*, F33.*, F34.1, F53.0). No exclusion for comorbid conditions, including other psychiatric conditions, was applied. The diagnosis codes used to identify depression patients do not include any codes for bipolar disorder; however, individuals with a diagnosis of bipolar disorder were not excluded from the study if they satisfied the definition of depression.

The index date was the first observed medical claim in the database with a diagnosis of depression, resulting in a cohort of newly diagnosed depression patients. Patients were required to have continuous enrollment in the database at least 1 year prior to and 3 years following the index date. Patients were excluded if they had evidence of treatment for depression – with an antidepressant or another treatment class of interest as defined below – more than 30 days prior to index, as our goal was to include patients who were newly diagnosed with depression and identify their treatment patterns from the beginning.

### Treatments and sequencing

**Fig. 1:**
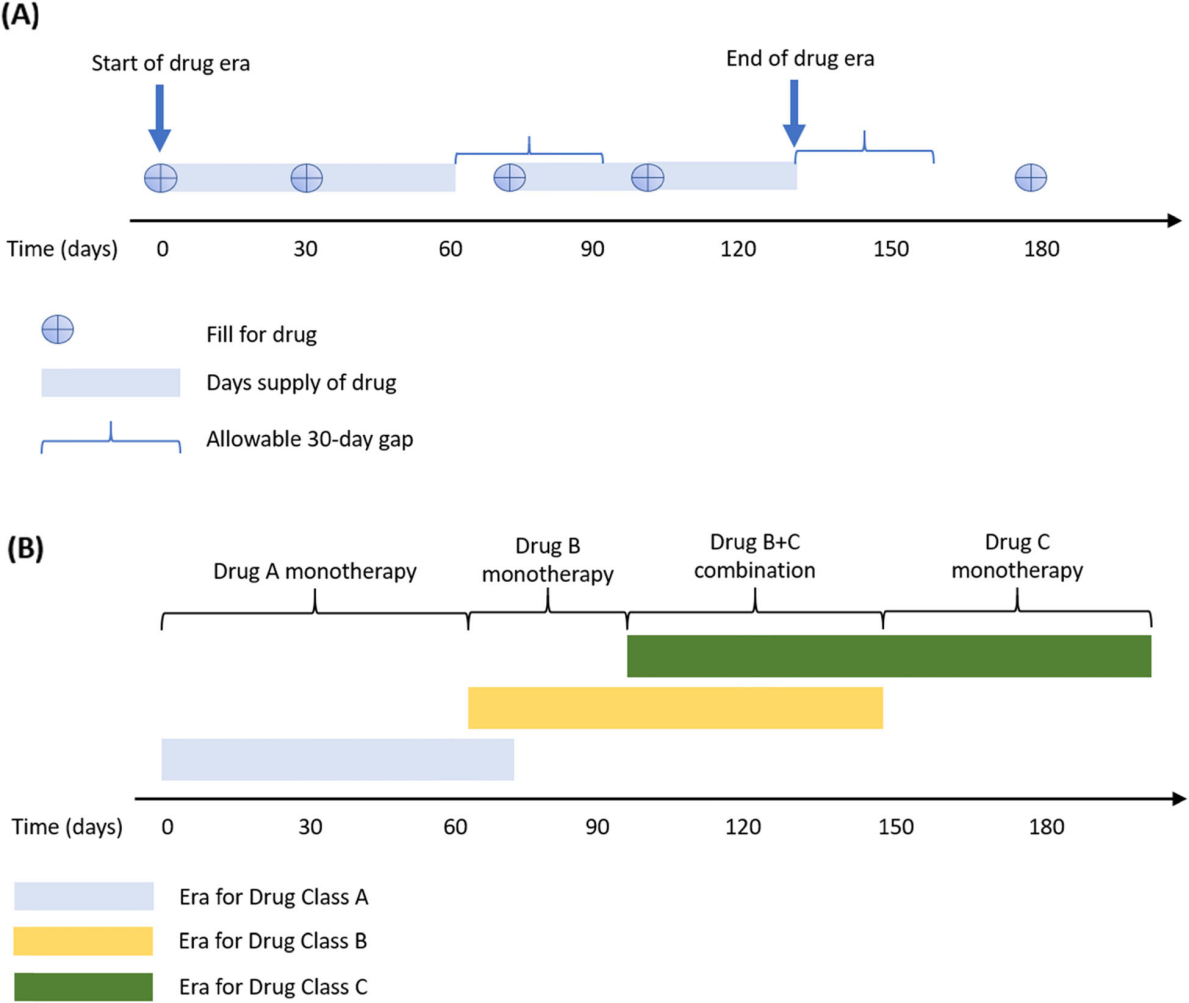
Study design illustration for drug eras, switching and combination therapy classification. **a** Drug eras are illustrated assuming a 30-day supply for each medication fill and allowing for a 30-day gap between the end of supply and the next fill. The drug era ends if another fill is not received within this gap. **b** If drug eras of two classes overlap at least 30 days (Drug Class B and Drug Class C) then it is classified as combination therapy, otherwise it is a switch between two classes (Drug Class A to Drug Class B)

Treatment patterns were captured at the class level and included SSRIs, serotonin and norepinephrine reuptake inhibitors (SNRIs), tricyclic antidepressants (TCAs), monoamine oxidase inhibitors (MAOIs), other antidepressants (including bupropion and trazodone, among others), anxiolytics, hypnotics/sedatives, antipsychotics, psychostimulants and lithium. The individual drugs included in each class and their corresponding RxNorm ingredient codes used to identify them from the databases are found in the Appendix. Treatment sequences were captured during 30 days prior to the depression index date through all available follow-up, a minimum of 3 years. The term “treatment line” is used to describe the sequence of medication classes and combinations of medication classes received by patients during this time. Use of a specific medication class was captured at the first instance and not counted again in later lines of therapy – for example an individual filling an SSRI, switching to an SNRI, and then moving back to an SSRI would only be captured as switching from SSRI to SNRI. Because the analysis is at the class-level, in-class switching and in-class combination therapy is not captured. Drug eras were calculated as the time from the first fill for a drug in a medication class until discontinuation of that medication, allowing for gaps of up to 30 days beyond the days supply of a prescription (Fig. 1). Combination therapy with multiple classes was defined as having at least 30 days of overlap in drug eras of more than one treatment class. A fill for a medication following discontinuation of a previous drug or with fewer than 30 days of overlap was considered a switch.

### Data source

The analysis was executed in four US-based administrative claims databases. Each database contains data from adjudicated health insurance claims (e.g., inpatient, outpatient/emergency department, and outpatient pharmacy) and health plan enrollment information. Briefly, the four databases included in this study were:

1. IBM MarketScan® Commercial Database (CCAE): Includes data from 142 million individuals enrolled in employer-sponsored insurance health plans. At the time of this study data were available from January 1, 2000 through January 31, 2019.
2. IBM MarketScan® Multi-State Medicaid Database (MDCD): A claims database for 26 million Medicaid enrollees from multiple states. At the time of this study data were available from January 1, 2006 through June 30, 2018.
3. IBM MarketScan® Medicare Supplemental Database (MDCR): Includes data for more than 9 million retirees with primary or Medicare supplemental coverage through privately insured fee-for-service, point-of-service, or capitated health plans. At the time of this study data were available from January 1, 2000 through January 31, 2019.
4. Optum© De-Identified Clinformatics® Data Mart Database. Includes 84 million members with private health insurance, who are fully insured in commercial plans or in administrative services only and Medicare Advantage (Medicare Advantage Prescription Drug coverage. The population is representative of US commercial claims patients (0–65 years old) with some Medicare (65+ years old). At the time of this study data were available from May 31, 2000 through December 31, 2018.

Data elements included were outpatient pharmacy dispensing claims (coded with National Drug Codes), inpatient and outpatient medical claims which provide diagnosis codes (coded in ICD-9-CM or ICD-10-CM) associated with a visit. The use of the IBM MarketScan and Optum claims databases was reviewed by the New England Institutional Review Board (IRB) and was determined to be exempt from broad IRB approval, as this research project did not involve human subjects research.

### Baseline characteristics

The mean age of patients (and standard deviation) on the index date within each data source, and the proportion of female patients were calculated. Comorbid conditions and the Charlson Comorbid Index [9] were captured during the year preceding, which included the index date. One diagnosis code for the comorbidity of interest was required during this time frame. Comorbidities were defined according to the Systematized Nomenclature of Medicine - Clinical Terms (SNOMED CT) classification system. The SNOMED CT classification allows mapping of various diagnostic languages across more than 80 countries, including, for example, ICD-9-CM, ICD-10-CM, and Read codes, to a single standardized set of concepts, and is used by the common data model leveraged for this study, described in the next section [10, 11]. Prevalence of all conditions defined by SNOMED were calculated, but only those that were most common across multiple databases and/or of special interest are reported. No statistical testing was performed to test for differences between data sources because with such a large number of patients even very small differences will be statistically significant at *p* < 0.05, thus the more important inference is the qualitative difference between groups.

### Common data model

Data from all four databases were mapped to standard concepts according to the Observational Medical Outcomes Partnership (OMOP) Common Data Model v5.0 [12] and the treatment sequence analysis was performed within the Observational Health Data Sciences and Informatics (or OHDSI, pronounced “Odyssey”) framework. Use of the common data model allowed for consistent and precise replication across each of the four databases.

## Results

A total of 269,668 patients were included from across the four databases (Table 1). The databases represent a wide range of ages, with the youngest patients from the CCAE and MDCD, while older individuals are more heavily represented in the MDCR and Optum databases. Females accounted for 62% of the patient population. Comorbid conditions such as pain, inflammatory disorders, hyperlipidemia, hypertension and diabetes were common in this population, particularly in the older Medicare population. A prior history of drug dependence was more common in the Medicaid patients (15.6%) compared with the other databases (6.5–10.2%), and prior evidence of suicidal thoughts was found in more than 1 in 25 Medicaid patients versus the lowest prevalence of 1 in 100 individuals from Medicare. A diagnosis for other conditions associated with the treatment classes analyzed – including bipolar disorder, post-traumatic stress disorder, psychotic disorder, obsessive compulsive disorder, and personality disorder – were relatively uncommon with most occurring in fewer than 2% of patients. One notable exception to this is the proportion of patients with a previous diagnosis of psychotic disorder in the MDCD population (6.9%).

**Table 1:** Characteristics of patients from each of the four study databases

Characteristic	CCAE (*N* = 114,543)	MDCD (*N* = 69,006)	MDCR (*N* = 5660)	Optum (*N* = 80,459)
Age at index (years), Mean (SD)	32.4 (15.6)	37.9 (25.7)	74.5 (8.5)	51.0 (23.5)
Gender: Female, %	60.4%	65.6%	57.7%	62.4%
Charlson comorbidity index score^a^, Mean (SD)	0.5 (1.3)	1.9 (2.9)	2.9 (2.6)	1.7 (2.5)
Common comorbid conditions^a^				
Pain	46.6%	58.7%	68.8%	59.1%
Inflammatory disorder of the respiratory system	33.8%	38.2%	31.4%	33.4%
Anxiety disorder	25.7%	21.3%	19.8%	27.3%
Traumatic injury	21.6%	27.4%	30.8%	25.4%
Arthropathy	20.3%	29.8%	51.9%	35.9%
Dysthymia	20.3%	8.6%	17.4%	15.7%
Inflammatory disorder of digestive system	20.2%	24.1%	18.0%	19.1%
Neoplastic disease	14.8%	10.2%	43.6%	24.3%
Hyperlipidemia	13.2%	23.7%	58.0%	38.0%
Visual system disorder	12.7%	31.3%	52.8%	31.7%
Hypertensive disorder	12.7%	36.6%	67.6%	39.2%
Malaise and fatigue	12.3%	11.9%	22.5%	19.2%
Backache	10.8%	16.9%	20.1%	17.8%
Vascular disorder	8.1%	19.9%	45.9%	24.8%
Drug dependence	6.5%	15.6%	8.0%	10.2%
Osteoarthritis	5.5%	16.5%	34.1%	19.6%
Diabetes mellitus	5.0%	18.7%	26.7%	15.7%
Other conditions of interest				
Bipolar disorder	1.1%	4.3%	1.1%	1.5%
PTSD	1.8%	3.2%	2.2%	1.7%
Psychotic disorder	1.3%	6.9%	3.8%	2.7%
Obsessive compulsive disorder	0.8%	0.5%	0.5%	0.7%
Personality disorder	0.8%	1.7%	1.1%	0.9%
Suicidal history				
Suicidal thoughts	3.9%	4.3%	1.0%	2.9%
Suicidal deliberate poisoning	0.2%	0.2%	0.0%	0.2%

*Abbreviations*: *CCAE* IBM MarketScan® Commercial Database, *MDCD* IBM MarketScan® Multi-State Medicaid Database, *MDCR* IBM MarketScan® Medicare Supplemental Database, *SD* standard deviation

^a^The Charlson Comorbidity Index and individual comorbidities were captured during the 365 days preceding and including the index date

Roughly one-third of patients from the CCAE (29.5%), MDCR (33.5%), and Optum (35.9%) databases did not receive any antidepressant or related medication during the entire follow-up period, while more than half of patients in the MDCD data (51.9%) were untreated with pharmacotherapy (Table 2). Of patients who did receive a treatment, approximately half went on to receive a second treatment class (range across databases: 47.8–59.5%), more than a quarter received a third (25.0–31.6%), and more than one in ten received a fourth (10.3–15.7%).

**Table 2:** Proportion of patients who were untreated or received, at least one, two, three, or four distinct treatment lines during the entire follow-up period

	CCAE (*N* = 114,543)		MDCD (*N* = 69,006)		MDCR (*N* = 5660)		Optum (*N* = 80,459)	
Treatment line	% of all patients	% of treated	% of all patients	% of treated	% of all patients	% of treated	% of all patients	% of treated
Untreated	29.5%		51.9%		33.5%		35.9%	
1st	70.5%	100%	48.1%	100%	66.5%	100%	64.1%	100%
2nd	42.0%	59.5%	23.0%	47.8%	35.5%	53.4%	34.4%	53.7%
3rd	22.3%	31.6%	12.0%	25.0%	16.7%	25.1%	17.1%	26.7%
4th	11.1%	15.7%	5.9%	12.2%	6.8%	10.3%	7.8%	12.2%

*Abbreviations*: *CCAE* IBM MarketScan® Commercial Database, *MDCD* IBM MarketScan® Multi-State Medicaid Database, *MDCR* IBM MarketScan® Medicare Supplemental Database

The most common medication class used during first-line therapy was SSRI, however there was variability in their use – more than half of patients from the CCAE database received SSRI as monotherapy or part of a combination first line treatment (57.5%) compared with one-third of patients in the MDCD database (36.3%) (Table 3). Non-antidepressant use made up a significant share of first-line treatments, with anxiolytics as the next most common first line treatment class received in all databases except for MDCD where hypnotic/sedative use was found in 22.6% of patients. Use of antipsychotics was not an uncommon first line approach, nearly 12% of Medicaid patients and more than 5% of all other patients received an antipsychotic as their initial treatment. The higher prevalence of antipsychotic use in the MDCD population was likely due to a higher prevalence of comorbid psychotic disorders in this group.

**Table 3:** Proportion of patients treated with each medication class out of those receiving first-line therapy (includes mono- or combination therapy)

	CCAE (*N* = 80,810)		MDCD (*N* = 33,186)		MDCR (*N* = 3764)		Optum (*N* = 51,585)	
SSRI	46,432	57.5%	12,035	36.3%	1693	45.0%	25,942	50.3%
Anxiolytic	14,625	18.1%	6141	18.5%	695	18.5%	9095	17.6%
Other antidepressant	12,196	15.1%	3578	10.8%	518	13.8%	7438	14.4%
Hypnotic/Sedative	6981	8.6%	666	2.0%	99	2.6%	1218	2.4%
Anticonvulsant	7377	9.1%	1813	5.5%	514	13.7%	5855	11.4%
SNRI	4531	5.6%	966	2.9%	249	6.6%	3145	6.1%
Antipsychotic	4609	5.7%	2120	6.4%	275	7.3%	2944	5.7%
Tricyclic	1426	1.8%	842	2.5%	61	1.6%	926	1.8%
Stimulant	1048	1.3%	426	1.3%	19	0.5%	1537	3.0%
Lithium	246	0.3%	106	0.3%	2	0.1%	110	0.2%
MAOI	7	0.0%	2	0.0%	3	0.1%	16	0.0%

*Abbreviations*: *CCAE* IBM MarketScan® Commercial Database, *MDCD* IBM MarketScan® Multi-State Medicaid Database, *MDCR* IBM MarketScan® Medicare Supplemental Database, *SSRI* Selective serotonin reuptake inhibitor, *SNRI* Serotonin and norepinephrine reuptake inhibitor, *MAOI* Monoamine oxidase inhibitor

Combination therapy with at least two distinct medication classes was used as the first line of treatment for 15.4–20.4% of patients, and the prevalence of combination increased in later lines of therapy, approaching 40% by the fourth line (Table 4). Use of combination therapy was relatively similar across databases, though the commercially insured populations of Optum and CCAE had slightly higher use than the MDCD and MDCR populations.

**Table 4:** Prevalence of combination therapy during each treatment line within treated patients

Treatment line	CCAE	MDCD	MDCR	Optum
1st	20.4%	17.9%	15.4%	18.3%
2nd	32.7%	32.2%	30.7%	33.9%
3rd	36.1%	35.1%	32.8%	37.9%
4th	38.6%	38.6%	31.9%	40.0%

*Abbreviations*: *CCAE* IBM MarketScan® Commercial Database, *MDCD* IBM MarketScan® Multi-State Medicaid Database, *MDCR* IBM MarketScan® Medicare Supplemental Database

Within patients receiving monotherapy SSRI as their initial therapy, more than half of patients went on to receive a second treatment class (Table 5). Within these patients, combination of an SSRI plus another treatment class during second line occurred 27.2–31.9% of time time; however, use in combination with an antipsychotic – the only approved treatment class for adjunctive depression treatment – was found in a minority of patients (3.8–6.8%).

**Table 5:** Treatment patterns of those receiving first line monotherapy SSRI treatment

	CCAE		MDCD		MDCR		Optum	
Received first line SSRI (monotherapy)	34,453		8292		1341		19,588	
Patients on first line SSRI monotherapy who went on to receive second treatment class	19,231	55.8%	4812	58.0%	703	52.4%	10,090	51.5%
Second line is combination therapy	7814	40.6%	1784	37.1%	265	37.7%	4034	40.0%
Second line is combination therapy which includes an SSRI	6085	31.6%	1309	27.2%	224	31.9%	3200	31.7%
Second line is combination therapy of SSRI + antipsychotic	828	4.3%	325	6.8%	31	4.4%	385	3.8%

*Abbreviations*: *CCAE* IBM MarketScan® Commercial Database, *MDCD* IBM MarketScan® Multi-State Medicaid Database, *MDCR* IBM MarketScan® Medicare Supplemental Database, *SSRI* Selective serotonin reuptake inhibitor

**Fig. 2:**
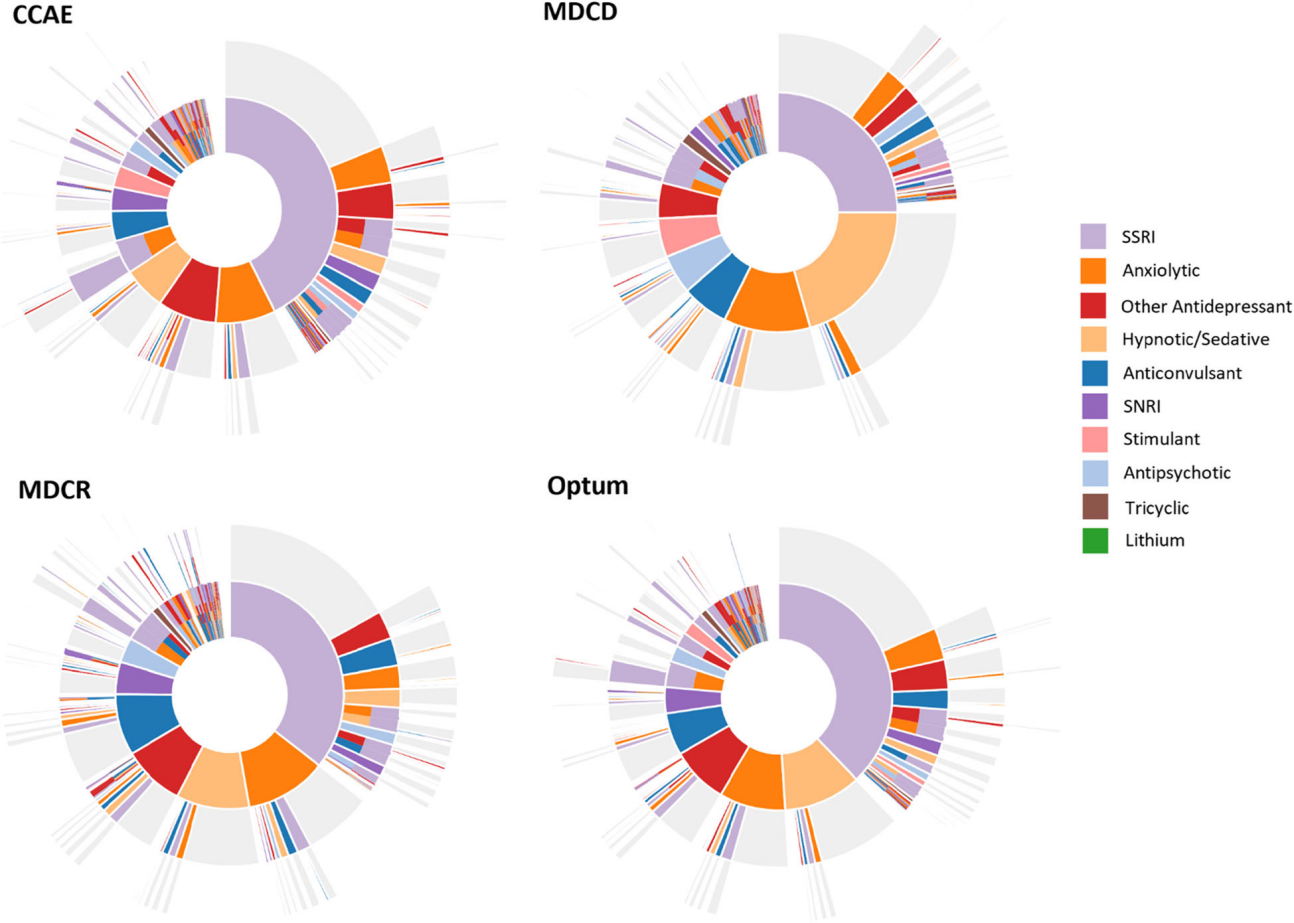
Sunburst of treatment patterns starting with first line (inner-most donut) to fourth line (outer slices). Each color represents a distinct treatment class, and each layer repesents a new treatment line and illustrate the sequence in which patients received different therapies; for example the large orange piece in the middle indicates first-line SSRI use, and the pink slice on the next outter ring adjacent to the orange indicates a switch from an SSRI to an anxiolytic. Slices that have multiple colors indicate combination therapy with more than one medication class. Slices in grey indicate no additional medication was taken. Abbreviations: CCAE, IBM MarketScan® Commercial Database; MDCD, IBM MarketScan® Multi-State Medicaid Database; MDCR, IBM MarketScan® Medicare Supplemental Database; SSRI, selective serotonin reuptake inhibitor; SNRI, serotonin and norepinephrine reuptake inhibitor

**Fig. 3:**
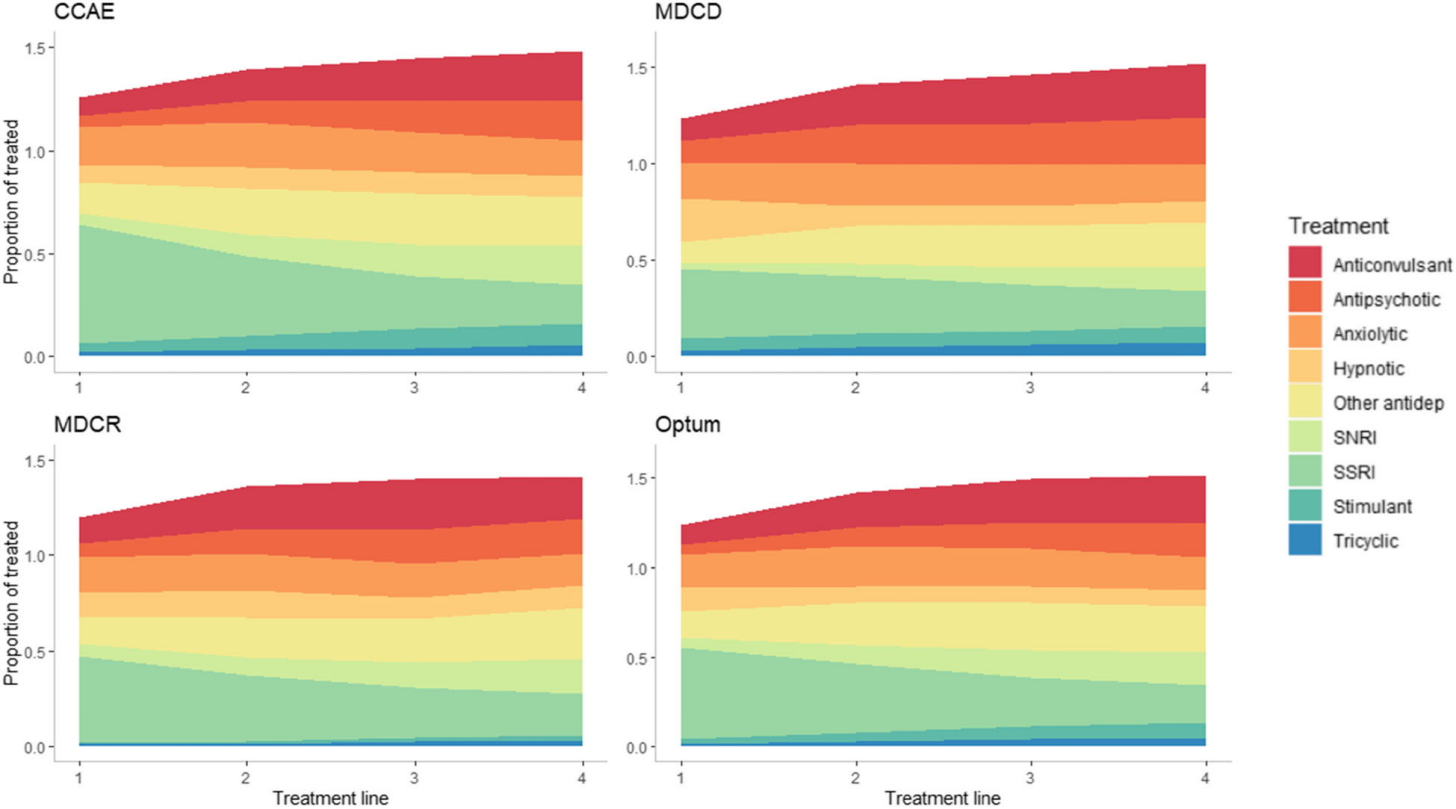
Proportional share of each treatment line by medication class. This area plot includes as the denominator individuals who received each line of therapy. The colors illustrate what share of treatment each of the different classes accounted for. Proportions go above 1.0 due to combination; individual medications that are part of a combination are counted in their respective medication class. Abbreviations: CCAE, IBM MarketScan® Commercial Database; MDCD, IBM MarketScan® Multi-State Medicaid Database; MDCR, IBM MarketScan® Medicare Supplemental Database; SSRI, selective serotonin reuptake inhibitor; SNRI, serotonin and norepinephrine reuptake inhibitor

The sequence of treatments within each database are shown in Fig. 2. This figure illustrates that while SSRI use was the most common first line treatment, the use of non-antidepressants – particularly anxiolytics, hypnotics/sedatives, and anticonvulsants – were common. Approximately half of patients starting on an SSRI never filled another class, while the other half moved on to a variety of different therapies. The second line therapies following first-line SSRI were not dominated by any single specific treatment class and include a mix of monotherapy treatments from other classes and the addition of a new treatments to an SSRI. The proportion of treatments accounted for by SSRIs decreased at each successive treatment line, while anticonvulsant, antipsychotic, and SNRI use steadily increases (Fig. 3); a pattern that was relatively consistent in each of the databases.

## Discussion

There has been limited prior research examining depression treatment sequences and the current work substantially expands upon this groundwork. Gauthier et al. [13] examined patterns of switches, combinations, dose escalation, and discontinuation of antidepressants in general, but did not look at individual drugs or classes, and did not include non-antidepressant treatment classes. Hripcsak et al. [14], used a similar methodology to identify depression treatment sequences in multiple databases; however, this analysis did not capture combination therapy or use of non-antidepressant medication classes, among other differences in the approach.

Our study leveraged prescription claims data from four patient populations representing a broad cross-section of the US population, including commercially insured individuals, those receiving Medicare, and individuals on Medicaid. There was a relatively low prevalence of first-line SSRI use (occurring in less than half of patients) in contrast to many of the treatment guidelines recommending starting with monotherapy SSRI [15, 16]. Furthermore, use of anxiolytics, anticonvulsants, and hypnotics/sedatives were commonly used as the first treatment choice in these patients newly diagnosed with depression, potentially pointing to a reluctancy of physicians to prescribe antidepressants [17] but being more comfortable using other classes of drugs such as anxiolytics [18, 19]. High use of benzodiazepines, which comprised the majority of anxiolytic use in this study, is concerning because they are not recommended as a first-line therapy [15] and they carry concerns of abuse [20–22] and risk of overdose [23, 24].

This study showed that while general trends across these populations were relatively similar there were some important differences. Specifically, patients covered by Medicaid tended to have treatment patterns that were different than the other three groups – more than half of patients diagnosed with depression were untreated, first-line SSRI use was lower, and use of alternative treatment classes outside of antidepressants occurred more often. The Medicaid sample represents a population of vulnerable individuals of lower socio-economic status and high burden of disease and it appears they are receiving different care when compared with the other patient populations.

The results of this real-world assessment of treatment practices appear to contradict some common treatment recommendations regarding treatment with pharmacotheapy. Many patients, ranging from one-third to one-half, received no pharmacotherapy for their depression during the entire follow-up, a period covering a minimum of 3 years in all patients. This was not limited to only Medicaid patients, as mentioned above, but also affected patients from the other databases. More so, this could be an underestimate of the true prevalence of untreated depression patients, because a significant proportion of individuals go undiagnosed and therefore are not able to receive treatment. Previous research screening individuals for depression rather than relying on a physician diagnosis has found that just 29–46% received a treatment for their depression [25, 26]. The results found in our study may be due to patients receiving alternative forms of treatment, such as psychotherapy, rather than pharmacologic treatment. It may also be that individuals who did not have dispensings had less severe depression or were prescribed non-pharmacological interventions. The American College of Physicians recommends clinicians choose between cognitive behavior therapy or second-generation antidepressants after discussing the pros and cons of the treatment choices with their patient [27]. And the American Psychiatric Association (APA) recommends psychotherapy alone as an initial treatment for patients with mild to moderate major depressive disorder [15].

The high prevalence of non-antidepressant treatment classes could reflect the high rates of comorbid conditions, such as anxiety disorder or sleep disorders [28]; however, these medications are largely being prescribed as monotherapy and not in combination with an antidepressant.

### Limitations

There are limitations to this study. This analysis focused only on pharmacotherapy for the treatment of depression and did not examine rates of psychotherapy or procedures such as ECT or TMS, which play an important role in the overall care patients receive, and may account for the proportion of patients that were classified as untreated. Patients with depression were identified using diagnoses codes which are not a perfect tool; however, we used a previously published algorithm for identifying depression in claims data which achieved high validity (PPV = 99%) [8]. Because the algorithm requires two outpatient or one inpatient diagnosis, there is less of a chance of falsely classifying a patient as having depression due to a rule-out or misdiagnosis that may happen if only requiring a single diagnosis. However, we do not capture depression patients who received only a single diagnosis of depression in an outpatient setting. This is a trade-off we deliberately made to improve certainty that we only included subjects with depression.

This analysis did not capture any within-class switching or combination; for example, receiving two SSRIs is simply captured as monotherapy SSRI use and switching from one SSRI to another does not appear as a change in therapy. This is because the goal of this study was to understand the order in which different therapy classes are first received over time, but this provides opportunity for future research to look in detail at the individual drug level to assess in-class treatment changes.

This study did not examine the average time patients were actively receiving each treatment, or how long patients may have been with no treatment between switching from one class to the next, as it was outside of the scope of this research. Discontinuation of antidepressant treatment is common and has been identified as a risk factor for relapses [29–33].

There is no diagnosis associated with prescription claims, thus receiving treatment for non-antidepressant classes is not guaranteed to have been prescribed for treating the underlying depression or its symptoms. To mitigate this misclassification due to receiving therapy for reasons unrelated to depression, we required treatment to occur at the time of or following the first diagnosis of depression with no prior history of treatment in the database; however, this does not guarantee that treatments could not have been for other conditions that began treatment following a patient’s first depression diagnosis. Additionally, the pharmacy claims are a record of medication dispensed to a patient, they do not capture prescriptions that were written by a physician but never filled by the patient.

Our study required three-years of continuous observation following the index depression diagnosis. This follow-up requirement was chosen to capture sufficient follow-up across the population to allow us to see multiple lines of therapy and various treatment changes. It’s possible, and even likely, that by doing so we are excluding a certain subset of individuals with depression, but the alternative of requiring too short of a follow-up period would have prevented us from seeing what happens during later lines of therapy and would distort the observed treatment patterns in the population as a whole. By making this decision we sacrificed broader generalizability of results but increased the validity of what was observed.

### Strengths

This study included more than a quarter-million individuals diagnosed with depression across four major claims databases representing a full-spectrum of ages and types of insurance coverage. When examined together, these databases provide generalizability to a broad cross-section of the United States. We were able to leverage the infrastructure of the common data model and the tools from the OHDSI network to achieve a uniform and consistent approach across each of these four databases whose underlying data structures differ. This work expands upon previous work by not limiting the analysis to only drugs that are specifically classified as antidepressants. It is widely known that medications in various other classes are commonly used to treat patients with depression and this study reflects real-world prescribing practices.

## Conclusions

This study provides the most detailed reflection of real-world pharmacotherapy practices for the treatment of depression in the United States to date. Understanding how depression patients are being managed is an important first step in improving care.

## Data Availability

Data are available on request.

## Abbreviations

APA: American Psychiatric Association
CCAE: IBM MarketScan® Commercial Database
ECT: Electroconvulsive therapy
ICD-10-CM: International Classification of Diseases, Tenth Revision, Clinical Modification
ICD-9-CM: International Classification of Diseases, Ninth Revision, Clinical Modification
IRB: Institutional Review Board
MAOI: Monoamine oxidase inhibitor
MDCD: IBM MarketScan® Multi-State Medicaid Database
MDCR: IBM MarketScan® Medicare Supplemental Database
OHDSI: Observational Health Data Sciences and Informatics
OMOP: Observational Medical Outcomes Partnership
SNRI: Serotonin and norepinephrine reuptake inhibitor
SSRI: Selective serotonin reuptake inhibitor
TCA: Tricyclic antidepressant
TMS: Transcranial magnetic stimulation
